# Effects of aging on ocular vestibular-evoked myogenic potential using ER-3A insert earphone and B81 bone vibrator

**DOI:** 10.3389/fneur.2022.956996

**Published:** 2022-08-25

**Authors:** Zhuo Xu, Zhilin Wang, Bo Zhong, Minjiao Wang, Xiaoqin Fan, Cuncun Ren, Meihao Qi, Ying Lin, Dingjun Zha

**Affiliations:** ^1^Department of Otolaryngology-Head and Neck Surgery, Xijing Hospital, Air Force Military Medical University, Xi'an, China; ^2^Division of Mechanics and Acoustics, National Institute of Metrology, Beijing, China

**Keywords:** oVEMP, aging, B81 bone vibrator, vestibular disorder, normal data

## Abstract

**Purpose:**

Aging is a process associated with degeneration and dysfunction of peripheral vestibular system or apparatus. This study aimed to investigate the influence of aging on ocular vestibular-evoked myogenic potential (oVEMP) response rates and recording parameters using the B81 bone vibrator and compare them with air conduction stimuli (ACS) oVEMP response characteristics.

**Methods:**

In 60 healthy participants aged 10–71 years (mean age 39.9; 29 male participants), the oVEMP response was elicited using a B81 bone vibrator and an ER-3A insert earphone. The effects of age and stimulus on oVEMP response rates and recording parameters were evaluated.

**Results:**

Response rates and amplitudes declined with aging using either ACS or bone-conducted vibration (BCV) stimulation, particularly in individuals over 60 years of age, whereas thresholds increased and N1 latencies were prolonged. BCV showed fewer risks of absent oVEMP response than ACS (*p* = 0.002). BCV acquired higher amplitudes (*p* < 0.001), lower thresholds, and shorter N1 and P1 latencies (all *p* < 0.001) than ACS.

**Conclusions:**

The absence of an oVEMP response may be attributed to aging rather than a concurrent vestibular disorder. B81-BCV likely produces higher mechanical drives to the vestibular hair cells at safer and non-traumatic levels compared with ACS and therefore may be more likely to evoke a response in the elderly cohort, whose vestibular function and mechanical sensitivity have declined. Thus, B81-BCV stimulation is more effective and safer to elicit oVEMPs, and it should be recommended when ACS fails in the clinic, particularly in the elderly population.

## Introduction

Aging causes continuous deterioration of human body, including the vestibular system (1, 2). Although the effects of aging on the functioning of the semicircular canals and saccule have been extensively investigated (3, 4), its impact on the utricle remains the least studied component.

Ocular vestibular-evoked myogenic potential (oVEMP), an excitatory electromyographic (EMG) response measured over the inferior oblique muscle that assesses contralateral utricular macula and superior vestibular nerve function, is increasingly being used in clinical practice (5, 6). The most commonly used clinical parameters of the oVEMP include response latency, amplitude, threshold, interaural amplitude asymmetry (IADR), and asymmetry ratio (AR). The oVEMP is reported to be helpful in the diagnosis of various vestibular disorders such as vestibular neuritis, Meniere's disease, and benign paroxysmal positional vertigo (7–11). Moreover, it is particularly significant in diagnosing third window disorder of the inner ear when combined with cervical vestibular-evoked myogenic potential which probes saccular macular function (12–14).

Ocular vestibular-evoked myogenic potentials can be elicited by a variety of stimuli, including air conduction stimuli (ACS) and bone-conducted vibration (BCV) stimuli, such as transient onset tone bursts or pulses, as well as galvanic stimulation (GVS) (5, 6, 15). However, when employing ACS oVEMP, the response rates and amplitudes decrease with aging and frequency tuning switches to higher frequencies (16–18), likely a feature of changes in morphology and mechanics, such as increased stiffness of the macula and otoconial layer. It was discovered that the absence of oVEMP responses was six times higher for those in their 40s, 50s, and 60s, and 13 times higher for people in their 70s than for those in their 20s (18). As a result, it is difficult to determine whether a missing ACS oVEMP response is related to vestibular disorders or whether ACS is an inefficient stimulus in the elderly population. Furthermore, in patients with middle ear disorder or pressure equalization tube dysfunction, ACS oVEMP may result in declined or absent responses. An air-bone gap of 9 dB nHL significantly reduces ACS VEMP responses (19), and an air-bone gap of 20 dB nHL completely abolishes ACS VEMP responses (20). Moreover, lower response rates for ACS oVEMP may be present in the ears following cochlear implantation due to air-bone gaps (21).

Bone-conducted vibration may be an alternative method to elicit oVEMPs, although the response rates of BCV oVEMPs can also be influenced by aging (15, 22). It has been reported that BCV may bypass the middle ear and be more effective in stimulating utricular neurons than ACS (23). This has been shown in mammalian models *in vivo*, where disrupting the ossicular chain and/or the conductive fluid layer between the stapes footplate and utricular macula abolished ACS utricular microphonics and vestibular short-latency-evoked potentials, but did not effect BCV-evoked responses (24). Furthermore, the use of a reflex hammer and lateral pulses (mini-shaker) in the interaural plane may be less affected by age assuming the receptor hair cells, and afferent neurons are functional (15). Other studies have demonstrated that bone-oscillating devices (Radioear B71) can elicit oVEMPs with higher response rates and amplitudes (15). Whereas BCV may be safer and provide more effective otolith organ stimulation than ACS, the B71 has been reported to be less dependable than the use of mini-shaker and impulse hammer in adults (25, 26). The Radioear B81 was recently designed to have a higher output and less distortion than traditional Radioear B71 (27). However, studies of normative data on the B81-BCV oVEMP responses are few, particularly in elderly Asians. Therefore, this study aimed to investigate the influence of aging on oVEMP response rates and recording parameters using the B81 bone vibrator and compare them with ACS oVEMP response characteristics. We hypothesized that aging might result in the decline of ACS and BCV induced oVEMP responses in healthy participants. Moreover, the response rates and amplitudes of the oVEMP would be higher for B81-BCV than for ACS, particularly in elderly individuals.

## Materials and methods

### Participants

A total of 60 healthy participants ranging from 10 to 71 years old (mean age 39.9 years; 29 males) were sorted into six age groups based on the decades of life. Individuals with a history of known conductive hearing loss (CHL), dizziness, balance symptoms, high blood pressure, diabetes, and neurologic illnesses were excluded. All participants underwent pure-tone audiometry and immittance assessment before oVEMP testing to rule out a conductive pathology. Those showing “A” type tympanogram were included in this study. The participant's hearing was considered normal if the pure-tone average (PTA) was ≤20 dB nHL. Participants were excluded if they had a CHL, defined as an air-bone gap >10 dB nHL at two consecutive frequencies. Symmetrical sensorineural hearing loss (SNHL), PTA ≤35 dB nHL, was permissible in participants aged >60 years. Participants with asymmetric oVEMP responses, with an IADR >0.33, were excluded for possible unilateral vestibular system impairment (18). This study was approved by the Medical Ethics Committee of the First Affiliated Hospital of the Air Force Medical University (No. KY20222045-C-1), and the informed consent was obtained from each participant.

### oVEMP testing

The oVEMP responses were acquired using the Interacoustics Eclipse EP1/25 System (Interacoustics, Middelfart, DK) in a conventional sound isolation room. Participants were instructed to lie down comfortably on an examination bed and avoid any extraneous movements of the body, limb, or neck. The electrode configuration included the placement of the active electrode approximately 1 cm below the lower eye-lid closest to the inferior oblique when staring up, the reference electrodes cross-posted 2 cm below the active electrode, and the ground electrode on the forehead, as found to be optimal and used previously (4, 28). Before electrode installation, the skin overlying the electrode locations was cleaned with a commercially available abrasive skin preparation gel to reduce electrode impedance. Using commercially available conductive paste, gold-plated cup-shaped electrodes were inserted at these spots and fastened with adhesive tape. This was important to ensure that altered oVEMP amplitudes were not due to the factors related to the electrode. The absolute and interelectrode impedances were maintained below 5 and 2 kΩ, respectively. The parameter settings of oVEMP are shown in Table 1, as we previously depicted (29).

**Table 1:** Parameter setting of oVEMP in this study.

**Parameter type**	**Parameter**
Stimulus	500 Hz tone burst
Stimulus type	Tone burst: 1–2–1 ms
Stimulus rate	5.1/s
Filters	1~1,000 Hz
Recording epoch	50 ms
Repetition	80
Gain	20,000

Air conduction stimuli and BCV oVEMP testing were performed using an ER-3A insert earphone and B81 bone vibrator (Radioear, USA), respectively. A B81 bone vibrator was placed on the mastoid using a standard bone conduction headband. Participants were asked to focus their gaze upward by 30° on a target attached to the wall during the recording. The stimulus intensity used by tone bursts of 500 Hz was 100 dB nHL for ACS and 70 dB nHL for B81-BCV. The outcomes of the oVEMPs were recorded from the contralateral ocular muscles during the initial stimulus intensity. After eliciting a typical waveform, the stimulus intensity was reduced in 5-dB decrements until no response was elicited. The threshold was the minimum stimulus intensity required to elicit reproducible oVEMP responses. We measured the output intensity of the ER-3A insert earphone and the B81 using a common calibration system, as previously reported (29, 30). For bone-conducted force calibration, an artificial mastoid (model 4930, Bruel & Kjaer, DK) was used as a transducer. The analysis was carried out using a data acquisition system (model 3160, Bruel & Kjaer, DK) and PULSE software package (version 20, Bruel & Kjaer, DK). For ACS, the measured peak-to-peak equivalent sound pressure level (peSPL) is referenced to 20 μPa of the air-conduction sound level, whereas for BCV, the measured peak-to-peak vibratory force level (peVFL) is referenced to 1 μN of the vibratory force level. The human mastoid is not as simple as an artificial mastoid concerning mechanical point impedance. Therefore, the position of the vibrator will be adjusted repeatedly to obtain the best response, considering input force and response sensitivity.

**Figure 1:**
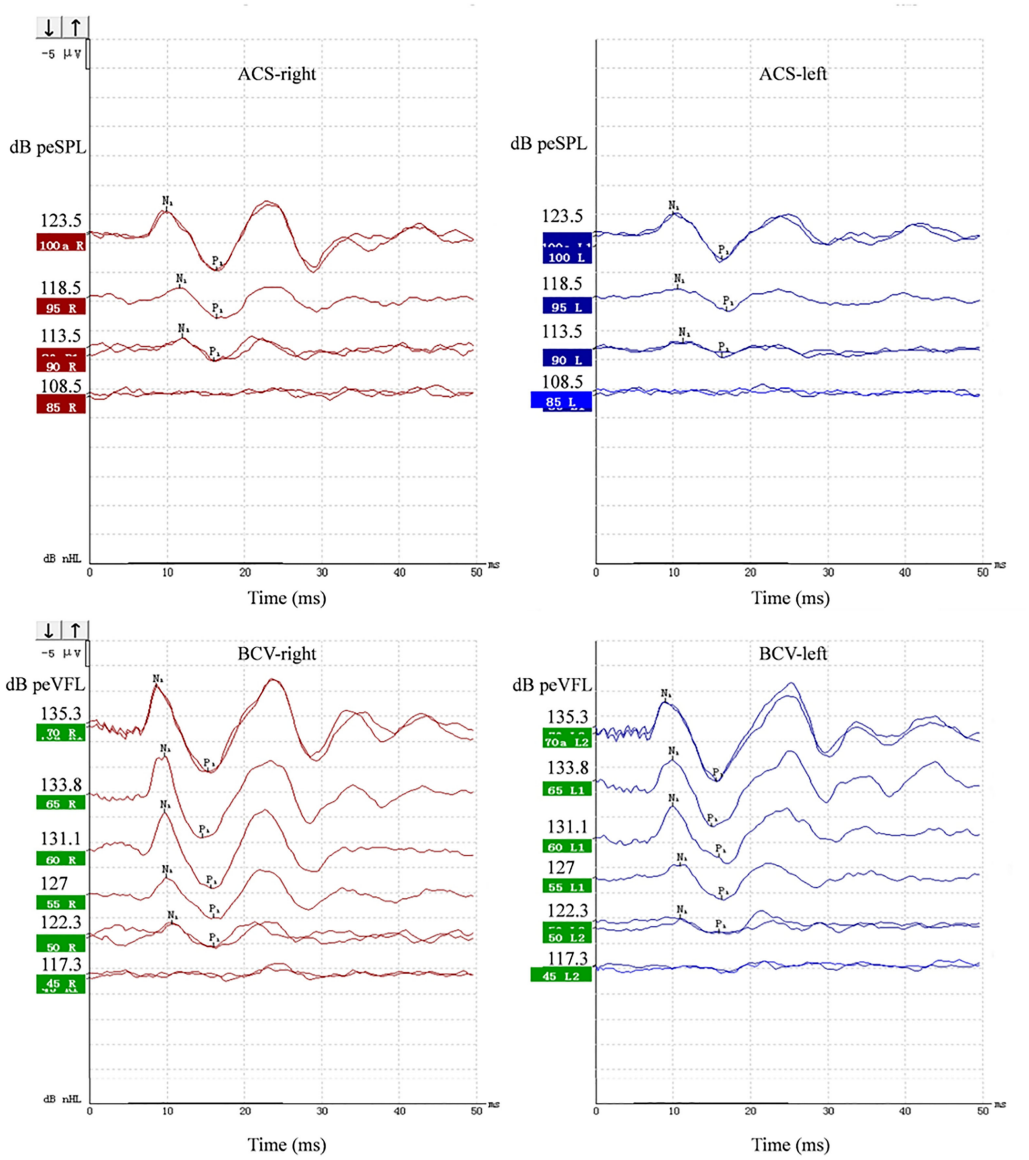
Representative ACS and BCV oVEMPs from a 19-year-old female. Primitive test value of tone burst parameter and corresponding sound pressure level or force level value were all marked on the *Y*-axis of each wave. The unit was peak-to-peak equivalent sound pressure level (peSPL) when using ACS and peak-to-peak force equivalent vibratory force level (peVFL) when using BCV.

The typical oVEMP is a biphasic waveform. The first wavepeak is the N1 with a latency of approximately 10 ms, followed by the P1 at ~15 ms. Figure 1 depicts a healthy participant's representative oVEMP tracings for ACS and BCV stimuli. If the oVEMP N1-P1 differed in latency from the typical 10–15 ms, then it was only acceptable if it deviated by several milliseconds, between 8 and 22 ms. Outcome measures for oVEMP testing included the N1 and P1 latencies, N1–P1 amplitudes, thresholds, IADR, and AR. The IADR and AR between a subject's ears were calculated using the following formula (31):

$$\begin{array}{l} \text{IADR}=\frac{\text{Left amplitude}}{\text{Right amplitude}}\\ \text{ AR}=|\frac{\text{Left amplitude}-\text{Right amplitude}}{\text{Left amplitude}+\text{Right amplitude}}| \end{array}$$

### Statistical analysis

Pearson's chi-square test and Fisher's exact test were used to compare the response rates of oVEMPs between the stimulus and age groups. Logistic regression analysis was performed to determine the effects of age (continuous), sex, side, and stimulus on absent oVEMP responses. Spearman's correlation analysis was used to show the relationship between age and recording parameters of the oVEMP. A linear mixed-effects model was employed to model the recording parameters of oVEMP, and the model was adjusted for sex, age (continuous), stimulus, and side. All statistical analyses were performed using SPSS v.24.0 (SPSS, Inc., Chicago, IL, USA). Differences were considered statistically significant if the *p*-value was <0.05.

## Results

### Effects of aging and stimulus on response rates of oVEMPs

**Figure 2:**
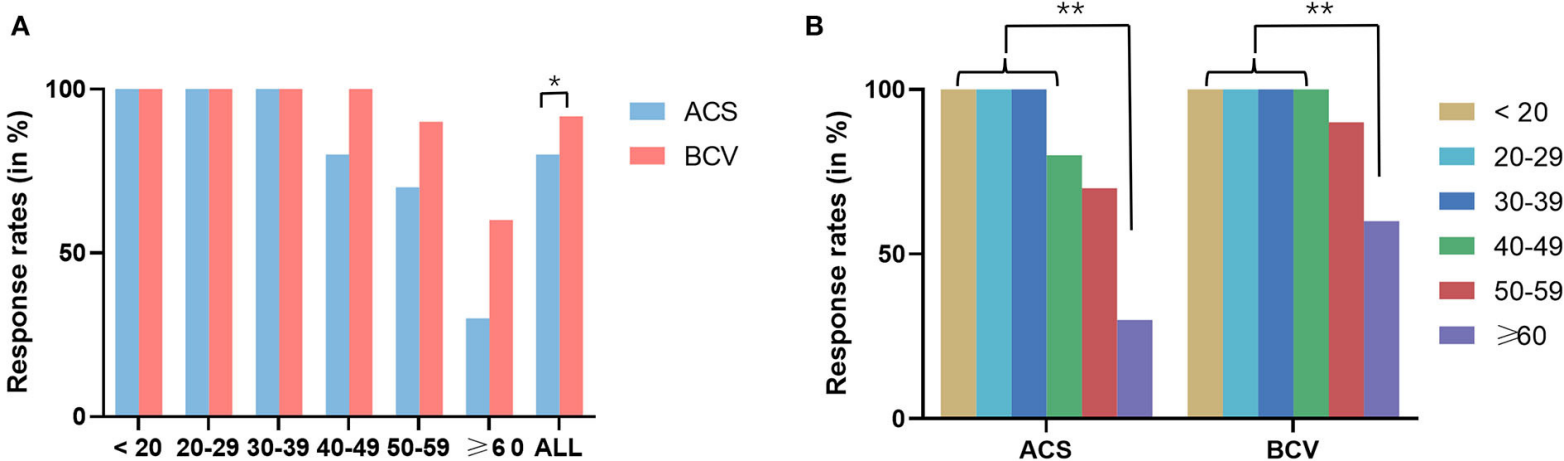
**(A)** Comparison of response rates between ACS and BCV oVEMPs in different age groups using Pearson's chi-square test and Fisher's exact test. **(B)** Comparison of response rates between different groups of age in ACS and BCV using Fisher's exact test with Bonferroni adjusted multiple comparisons for pair-wise comparisons. Star-marked comparisons are statistically significant at *p < 0.05 and **p < 0.01.

The demographics and response rates of oVEMPs for each stimulus across different age groups are shown in Table 2. The oVEMPs were present in both ears of all participants up to the age of 40 for ACS and 50 for BCV. There was no statistically significant difference in response rates between the ACS and BCV groups in the age groups of 40–49 years (Fisher's exact test, *p* = 0.106), 50–59 years (Fisher's exact test, *p* = 0.235), and ≥60 years (χ^2^ = 3.64, *p* = 0.057). However, the BCV group showed significantly higher response rates than the ACS group (χ^2^ = 6.72, *p* = 0.010), considering all patients (Figure 2A).

**Table 2:** Demographics and response rates of oVEMP for each stimulus across the age groups.

**Age groups**	***N***	**Mean age (SD)**	**Sex (M, F)**	***N*** **(response)**		**Response rates (in %)**	**χ^2^**	***p***
				**Left**	**Right**			
<20	10	15.2 (3.40)	4, 6					
ASC				10	10	100		
BCV				10	10	100		CNP
20~29	10	25.5 (2.26)	6, 4					
ASC				10	10	100		
BCV				10	10	100		CNP
30~39	10	34.2 (2.86)	6, 4					
ASC				10	10	100		
BCV				10	10	100		CNP
40~49	10	44.7 (2.34)	4, 6					
ASC				8	8	80		
BCV				10	10	100	–	0.106
50~59	10	53.2 (1.93)	4, 6					
ASC				7	7	70		
BCV				9	9	90	–	0.235
≥60	10	66.6 (3.27)	5, 5					
ASC				3	3	30		
BCV				6	6	60	3.64	0.057
ALL	60	39.9 (17.49)	29, 31					
ASC				48	48	80		
BCV				55	55	91.7	6.72	0.010

ACS, air conduction stimuli; BCV, bone-conducted vibration; CNP, could not be performed. –, Fisher's exact test.

In both the ACS and BCV groups, Fisher's exact test with Bonferroni-adjusted multiple comparisons for pair-wise comparisons across different age groups found no significant difference in response rates between the age groups up to 60 years, as shown in Figure 2B. However, the age groups up to 50 years showed significantly higher response rates than those older than 60 years for both ACS and BCV (*p* < 0.001). The age group of 50–60 years showed no statistically significant difference in response rates compared with the age groups beyond 60 years for both ACS and BCV.

The logistic regression model with the enter method was statistically significant in determining the effects of age (continuous), sex, side, and stimulus on an absent oVEMP response (χ^2^ = 28.63, *p* < 0.001). As shown in Table 3, the model was adjusted for sex, age (continuous), and stimulus. Men had a greater risk of absent oVEMP responses than women (OR = 2.607). BCV showed fewer risks of absent oVEMP response than ACS (OR = 0.210). Furthermore, the risk of the absence of an oVEMP response increased with age (OR = 1.144).

**Table 3:** Logistic regression analysis to evaluate factors with odds ratios (95% confidence interval) for absent oVEMP responses.

**Factor**	***B***	**Wald**	***p***	**Odds ratios**	**95%CI**
Sex	0.958	3.894	0.048	2.607	1.007, 6.750
Side	0	0	1	1	0.394, 2.535
Age	0.134	35.051	<0.001	1.144	1.094, 1.196
Stimulus	−1.562	9.260	0.002	0.210	0.077, 0.574

CI, confidence interval; sex (0 = female, 1 = male), stimulus (0 = ACS, 1 = BCV).

### Effects of aging and stimulus on recording parameters of OVEMPs

**Figure 3:**
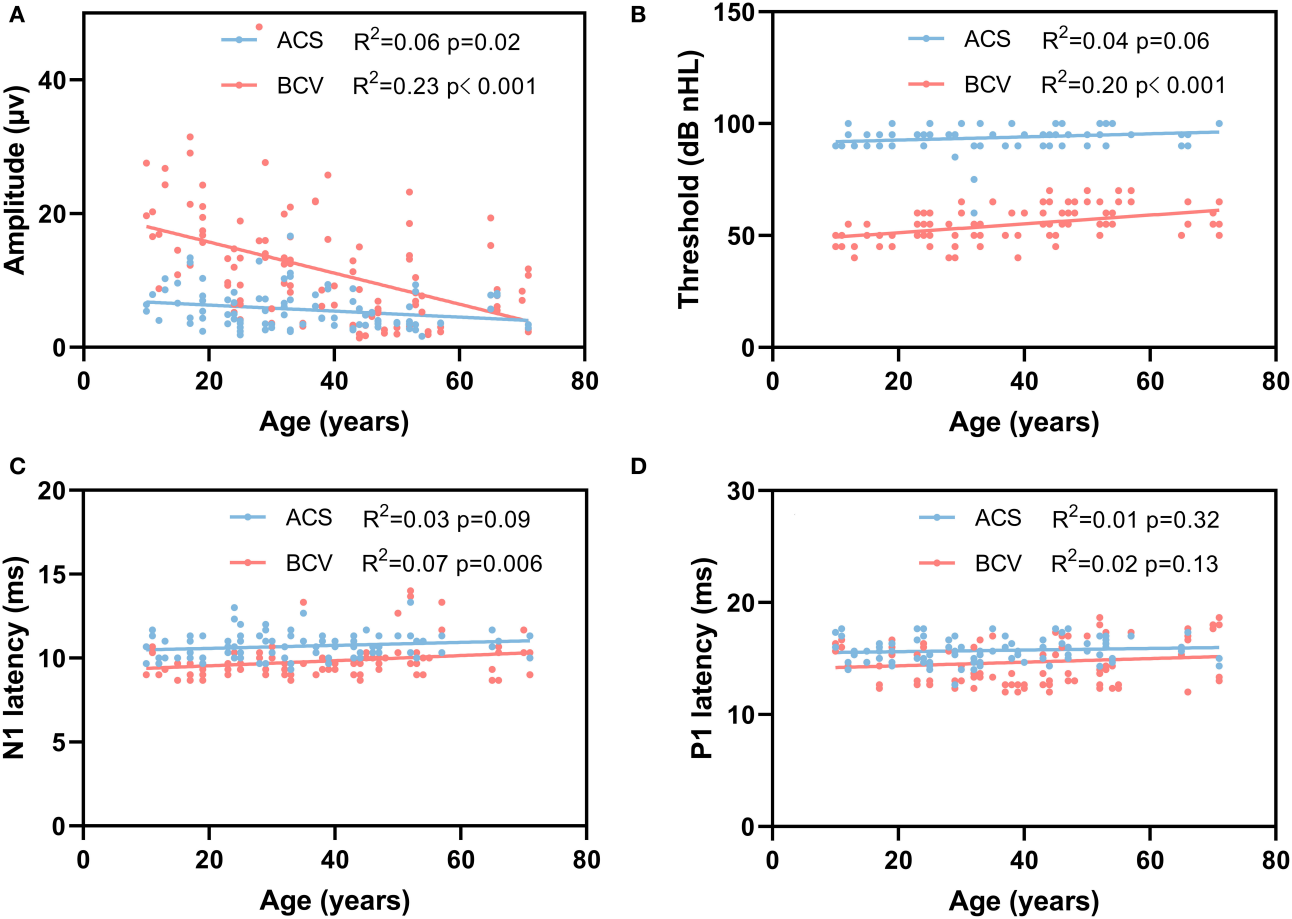
Simple linear regression curves showed the relationship between age and oVEMP recording parameters. **(A)** A significantly negative correlation between age and amplitude for ACS (*p* = 0.02) and BCV (*p* < 0.001). **(B)** A significantly positive correlation between age and thresholds for BCV (*p* < 0.001). **(C)** A significant positive correlation was found between age and N1 latency for BCV (*p* = 0.006). **(D)** There was no correlation between the age and P1 latency for ACS and BCV.

The recording parameters of the oVEMP for each stimulus across different age groups are shown in Table 4. Figure 3 shows the linear regression curves depicting the relationship between age and the recording parameters. The results revealed a significantly negative correlation between age and amplitude for both ACS (*R*^2^ = 0.06, *p* = 0.02) and BCV (*R*^2^ = 0.23, *p* < 0.001) (Figure 3A). The correlation between age and thresholds was significantly positive for BCV (R^2^ = 0.20, *p* < 0.001), whereas not for ACS (Figure 3B). A significant positive correlation was found between age and N1 latencies for BCV (*R*^2^ = 0.07, *p* = 0.006), with no correlation for ACS (Figure 3C). Age showed no significant correlation with P1 latencies (Figure 3D), IADR, or AR for both ACS and BCV.

**Table 4:** Recording parameters of oVEMP for each stimulus across the age groups.

**Age groups**	**Ears (*N*)**	**Amplitude (uV)**	**Threshold (dB nHL)**	**N1 latency (ms)**	**P1 latency (ms)**	**IADR**	**AR**
**<20**							
ASC	20	7.08 (2.44)	92.25 (2.93)	10.38 (0.56)	15.73 (0.77)	1.06 (0.20)	0.11(0.07)
BCV	20	19.75 (4.84)	48.25 (3.43)	9.43 (0.48)	15.21 (0.89)	1.14 (0.27)	0.12(0.08)
**20~29**							
ASC	20	5.17 (2.05)	94.75 (2.40)	10.93 (0.73)	15.63 (1.03)	0.93 (0.28)	0.16 (0.06)
BCV	20	13.70 (5.92)	52.25 (5.75)	9.66 (0.34)	14.27 (1.53)	1.17 (0.30)	0.11 (0.07)
**30~39**							
ASC	20	6.62 (2.87)	91.25 (6.00)	10.51 (0.68)	15.40 (0.71)	0.91 (0.17)	0.11 (0.07)
BCV	20	12.61 (5.59)	54.25 (5.40)	9.63 (0.56)	13.85 (1.17)	0.94 (0.23)	0.12 (0.07)
**40~49**							
ASC	16	4.42 (1.25)	94.96 (1.78)	10.77 (0.42)	15.91 (1.01)	0.98 (0.23)	0.12 (0.06)
BCV	20	5.76 (2.30)	59.00 (4.80)	9.70 (0.40)	14.05 (1.56)	1.02 (0.26)	0.13 (0.09)
**50~59**							
ASC	14	4.12 (1.63)	96.07 (3.37)	10.97 (0.55)	15.97 (0.79)	0.98 (0.18)	0.10 (0.08)
BCV	18	7.45 (4.75)	59.44 (6.11)	10.66 (1.33)	15.07 (2.01)	1.05 (0.24)	0.10 (0.06)
**≥60**							
ASC	6	5.95 (1.94)	95.00 (3.330	10.95 (0.41)	15.94 (0.94)	1.17 (0.41)	0.08 (0.04)
BCV	12	8.79 (3.65)	56.67 (4.72)	9.89 (0.80)	16.11 (1.82)	0.96 (0.13)	0.07 (0.02)
**ALL**							
ASC	96	5.64 (2.94)	93.70 (5.58)	10.71 (0.78)	15.72 (1.08)	0.99 (0.28)	0.12 (0.09)
BCV	110	11.60 (8.09)	54.77 (7.31)	9.81 (0.99)	14.64 (1.85)	1.05 (0.34)	0.13 (0.10)

ACS, air conduction stimuli; BCV, bone-conducted vibration; AR, asymmetry ratio; IADR, interaural amplitude asymmetry.

Table 5 shows the results of multiple linear regression analysis using a stepwise method. The model was adjusted to evaluate the effects of sex, age (continuous), stimulus, and side on the oVEMP recording parameters. The results indicated that age was positively correlated with thresholds (*p* < 0.001) and negatively correlated with amplitudes (*p* < 0.001) and N1 latencies (*p* = 0.001). BCV oVEMP showed higher amplitudes, lower thresholds, and shorter N1 and P1 latencies (all *p* < 0.001). Furthermore, sex, age (continuous), and stimulus methods were not significantly correlated with IADR and AR. Interesting, BCV oVEMP amplitudes were always larger compared with ACS stimulation, even after they have declined by >50% in the age cohort of 20–60 (refer to Table 4).

**Table 5:** Evaluating the effects of gender, age (continuous), stimulus, and side on oVEMP response parameters using multiple linear regression analysis.

**Parameters**	**Factor**	***B***	**95%CI**	***p***
Amplitude	Gender	–	–	–
	Side	–	–	–
	Age	−0.15	−0.20, −0.10	<0.001
	Stimulus	6.41	4.82, 8.00	<0.001
Threshold	Gender	–	–	–
	Side	–	–	–
	Age	0.14	0.09, 0.20	<0.001
	Stimulus	−39.35	−41.05, −37.64	<0.001
N1 latency	Gender	–	–	–
	Side	–	–	–
	Age	0.01	0.005, 0.02	0.001
	Stimulus	−0.94	−1.18, −0.69	<0.001
P1 latency	Gender	–	–	–
	Side	–	–	–
	Age	–	–	–
	Stimulus	−1.08	−1.51, −0.66	<0.001
IADR	Gender	–	–	–
	Age	–	–	–
	Stimulus	–	–	–
AR	Gender	–	–	–
	Age	–	–	–
	Stimulus	–	–	–

AR, asymmetry ratio; CI, confidence interval; IADR, interaural amplitude asymmetry; Stimulus (0 = ACS, 1 = BCV).

## Discussion

We explored the effects of aging on the oVEMP response rate and parameters using the B81 bone vibrator and compared them to the ACS oVEMP response characteristics in this study. Similar to ACS oVEMPs, we discovered that the response rates and amplitudes of the B81-BCV oVEMPs declined with aging, particularly in individuals over 60 years of age, whereas the thresholds and N1 latencies increased. Furthermore, B81-BCV showed higher oVEMP response rates, greater amplitudes, lower thresholds, and shorter N1 latencies than ACS did. As a result, B81-BCV would be less susceptible to aging than ACS, which is consistent with our hypothesis. Owing to better response rates and lower thresholds, B81-BCV might be more effective and safer than ACS in eliciting oVEMPs.

### Effects of aging on oVEMPs response

Table 6 summarizes many studies conducted to investigate the impact of aging on oVEMPs. Owing to a lack of accessible information, we did not assess non-English publications in this area. Despite the variances in stimulators, most investigations have revealed a decrease in oVEMP response rates and amplitudes with age. These results are consistent with the discovery of age-related degenerative alterations from the end organs of the vestibular system to its central nuclei (37). Previous studies have shown that the mass of the utricular macula decreases and the stiffness of the utricular membrane increases as a result of degeneration associated with aging (38, 39), which therefore requires higher ACS or BCV drive to activate the vestibular hair cells. These degenerative alterations would have resulted in a decrease in amplitudes for the already small-amplitude oVEMP curves in certain people, resulting in merging of the response in the EMG noise. As a result, these responses would have been unidentified, resulting in lower oVEMP response rates in the elderly (4). This study appears to be consistent with earlier findings (4, 15, 18, 22, 31–36). However, those studies showed different onset age of reductions in oVEMP response rates and amplitudes. These differences may be attributed to the stimulator, sample size, and race of the participants.

**Table 6:** Literature review of the effects of age on oVEMP response rates and response parameters using ACS and BCV.

**Study**	**Sample** **size**	**Stimulator**	**Effect of age on oVEMP response parameters (beginning age in years)**						
			**Response rate**	**Threshold**	**Amplitude**	**N1 latency**	**P1 latency**	**IADR**	**AR**
Nguyen et al. (31)	53	ACS, foam eartips	NR	NR	>50	>50	>50	NR	UR
Rosengren et al. (15)	61	ACS, headphones BCV, B71	NR	↑	↓	↑	NR	NR	NR
Tseng et al. (32)	70	BCV, electromechanical vibrator	>60	NR	>60	>50	>50	NR	NR
Chang et al. (33)	69	BCV, electromechanical vibrator	>60	NR	>50	>50	>50	NR	NR
Piker et al. (17)	297	ACS, ER-3A insert earphone	>40	NR	↓	NR	NR	NR	NR
Versino et al. (34)	54	ACS	NR	NR	↓	UR	NR	NR	UR
Kumar et al. (35)	90	ACS, ER-3A insert earphone	NR	NR	>60	>60	>60	NR	NR
Li et al. (36)	257	BCV, reflex hammer	↓	NR	↓	↑	NR	NR	NR
Singh et al. (4)	480	ACS, ER-3A insert earphone	>50	NR	>50	>50	>50	UR	NR
Patterson et al. (22)	85	ACS, ER-3A insert earphone BCV, B81 and impulse hammer	↓	NR	↓	NR	NR	NR	NR

ACS, air conduction stimuli; AR, asymmetry ratio; BCV, bone-conducted vibration; IADR, interaural amplitude asymmetry. UR, unrelated; NR, not report; ↑, increase; ↓, decrease.

The largest sample study of aging on oVEMP response reported ACS oVEMP response rates of 41.25% in 80 participants aged >60 years, which is somewhat consistent with our study (4). Few studies have reported the effect of aging on the B81-BCV oVEMP response rates and response parameters (Table 6). Surprisingly, Patterson et al. reported oVEMP response rates of 92% for ACS and 83% for the B81 in the age of 60–69, which were greater than our response rates of 30 and 60%, respectively (22). This may have been due to the lower stimulation intensity of B81 with 70 dB nHL (135.3 dB peVFL) in our study, whereas Patterson et al. increased the stimulation intensity to 75dB nHL (138 dB peVFL) when oVEMPs could not be elicited with 70 dB nHL (136 dB peVFL) stimulation (22). In addition, BC sound transmission and its response potentials are affected by stimulation position as well as conditions and manipulations of the head (40). Another surprising finding was the effect of sex on response rates, wherein males demonstrated a greater risk of oVEMP response absence (OR = 2.607). To the best of our knowledge, no reports have indicated that sex influences oVEMP response rates. This sex difference may be due to the limitation of the sample size. The sample size was likely not representative of the general population for each age category, which needs to be expanded in further research.

Meanwhile, we discovered that older people required a more intense stimulus to elicit an oVEMP response, with longer N1 latencies. This is in line with the findings from earlier oVEMP research (15), which may also be explained by vestibular system degeneration (37). Accepting responses with peak latency a few milliseconds beyond the normal range, however, should be done with caution. Late peaks can also be caused by failure to manage the gaze angle (41) or insufficient stimulus intensity. Another finding in our study was that age did not affect IADR and AR, similar to the results of previous studies (4, 31). This may due to that aging is a symmetrical process that equally affects both sides of any two-sided system (42).

### ACS and B81-BCV

Our study discovered that the response rates of the B81-BCV oVEMPs were significantly higher than those of the ACS oVEMPs, especially in the elderly population >60 years. Here, the results reveal a 30% greater oVEMP response rate evoked by BCV compared to ACS. This may due to the potential differences in stimulation modes of peripheral receptor activation by ACS and BCV. Previous research has indicated that the saccule is more specific to ACS, whereas BCV stimulates both the saccule and utricle equally (43, 44). Pastras et al. (24) reported that during ACS stimulation, the fluid pressure wave coupling the stapes motion to the utricle primarily produces a transverse motion of the utricular macula, whereas BCV most likely induces a more complex motion of the utricular macula, where lateral motion of the macula may be the dominant drive activating the hair cells. Interestingly, for the same level of macular velocity, the utricular microphonic response amplitude was ~4 times larger during BCV compared to ACS, demonstrating the differences in sensitivity and micromechanical activation modes of utricular stereocilia between BCV and ACS in the mammalian labyrinth (24). These differences likely result in BCV producing higher mechanical input drives to the hair cells than ACS. These differences also likely exist in the human labyrinth, where BCV may be more effective in evoking oVEMPs in the clinic compared to ACS, for a similar level of macular stimulation. Moreover, B81-BCV is a bilateral stimulation of the utricle, with probably concurrent enhancement of the vertical component eye movement, interpreting the higher oVEMP response rates and amplitudes to BCV seen in this study (45).

Furthermore, the BCV oVEMP amplitudes are reduced by >50% from ages 20 to 60, whereas the ACS oVEMP amplitudes remain relatively low (Figure 3A), highlighting that BCV oVEMP amplitude is indeed affected by aging. Moreover, BCV oVEMP thresholds and N1 latency also slightly increase with aging more than ACS. The underlying mechanisms producing these differences are speculated that the mass of the utricular macula reduces and the stiffness of the utricular membrane increases with aging (38, 39). Additionally, the potential differences in stimulation modes of peripheral receptor activation by ACS and BCV likely also play an important role. Hence, the higher response rate for BCV in the elderly cohort is likely due to higher relative amplitudes of the BCV oVEMPs (even in the elderly, which is apparent in Figure 3A) and is likely also influenced by the limitation of using intense acoustic sounds to evoke ACS oVEMPs, which would damage the macular receptors.

Overall, there are some advantages of using BCV over ACS to elicit oVEMPs. First, in this study, the thresholds of BCV were significantly lower than ACS. It is of great significance for BCV to reduce the risk of damage from noise-related hearing loss due to lower thresholds. The high-intensity stimuli required for ACS VEMPs increase the risk of damage from sound pressure exposure in narrower ear canals, with ~3 dB nHL higher SPL in smaller ears (46, 47). Therefore, BCV may be employed instead of ACS to minimize the harm caused by loud stimuli. Second, for patients with CHL, such as middle ear or eustachian tube dysfunction, ACS may be an insufficient stimulus to stimulate the utricle in some individuals owing to attenuation during conduction, resulting in absent or diminished oVEMP response rates and amplitudes. BCV can directly stimulate the utricle and bypass the middle ear, avoiding attenuation of stimulation. Thus, compared to ACS, BCV offers a wider variety of applications. Finally, Merchant et al. reported that mechanical changes result in stiffening of the auditory system in patients with cochlear implantation (21). The response rates of BCV oVEMP were higher than that of ACS in these cochlear implant users, indicating that missing ACS responses were likely due to mechanical alterations rather than concurrent vestibular disorders.

### The limitation of B81-BCV

B81-BCV may be a more effective and safer stimulus than ACS in eliciting oVEMPs, especially in pediatric (probably with exudative otitis media) and elderly populations. Nevertheless, there are some limitations to B81-BCV. Declined or absent responses can be caused by small movements and variations in placement for the B81 (40, 48). Due to the limited force output of B71, forehead placement for B71 is not recommended for VEMPs (25). Although B81 has a higher output and less distortion than the B71, the B81 output is likely not strong enough for forehead placement (22, 48). Thus, we suggest mastoid placement for the B81. Variations in the mastoid anatomy and head movements may result in slight movement of the B81 bone vibrator. If the participant and examiner noticed movement during testing, the testing was repeated after adjusting the bone vibrator. However, it is not easy to accomplish the testing without any slight movements of B81 in people with large protruding mastoid processes. Reinforcing with tape may be helpful in reducing the movements required to accomplish testing. Moreover, the examiner should attempt to place it symmetrically on both sides to avoid position-related differences.

Bone-conducted vibration can result in complex harmonic distortions in the skull due to the distorted output from the vibrator/mini-shaker and/or the complex resonances in the cranium. It has been reported that the maximum output range with minimal to no harmonic distortion is between 120 and 128 dB peVFL (49), this will vary with subjects and stimulation parameters/type. Whatever, the stimulus intensity of the B81-BCV ranged from 117.3 dB peVFL to 135.3 dB peVFL in our study, which was outside the linear range. Besides, it has been reported that considering BCV-oVEMP is most sensitive to low-frequency stimulation, the energy at harmonic distortion products of 500 Hz may be unlikely to contribute to response generation (49).

## Conclusion

Aging affects oVEMP response rates and amplitudes regardless of stimulus type. Since B81 bone vibrator is more likely to evoke a response with lower intensity acoustic signal, it is more effective and safer than ACS to elicit oVEMPs, especially in the elderly population.

## Data availability statement

The raw data supporting the conclusions of this article will be made available by the authors, without undue reservation.

## Ethics statement

The studies involving human participants were reviewed and approved by Medical Ethics Committee of the First Affiliated Hospital of the Air Force Medical University (No. KY20222045-C-1). Written informed consent to participate in this study was provided by the participants' legal guardian/next of kin.

## Author contributions

YL and DZ designed the study. YL and ZX wrote the manuscript, performed the statistical analysis, and made graphs. BZ did the calibration of acoustic instrument. ZW, MQ, MW, XF, and CR did the data collection. All authors performed data interpretation and approved the manuscript.

## Funding

This research was supported by the grants from the National Key research and development program of China (2020YFC2005200) part 3 (2020YFC2005203), the National Natural Science Foundation of China under numbers 82171161 and 82101214, and Xijing Boost-Advanced Discipline Construction Project (XJZT19MDT02).

## Conflict of interest

The authors declare that the research was conducted in the absence of any commercial or financial relationships that could be construed as a potential conflict of interest.

## Publisher's note

All claims expressed in this article are solely those of the authors and do not necessarily represent those of their affiliated organizations, or those of the publisher, the editors and the reviewers. Any product that may be evaluated in this article, or claim that may be made by its manufacturer, is not guaranteed or endorsed by the publisher.
